# Inhibition of Histone Deacetylase by Butyrate Protects Rat Liver from Ischemic Reperfusion Injury

**DOI:** 10.3390/ijms151121069

**Published:** 2014-11-14

**Authors:** Jie Sun, Qiujv Wu, Huiling Sun, Yingli Qiao

**Affiliations:** 1 Department of Endocrinology, Liaocheng People’s Hospital, Liaocheng 252000, China; E-Mail: sunjie1982@126.com; 2 Department of Gastroenterology, Liaocheng People’s Hospital, Liaocheng 252000, China; E-Mails: q750592@126.com (Q.W.); sunhuiling1984@126.com (H.S.); 3 Department of General Surgery, Liaocheng People’s Hospital, Liaocheng 252000, China

**Keywords:** reperfusion injury, histone acetylation, histone deacetylase, butyrate

## Abstract

We showed previously that pretreatment of butyrate, which is an endogenous histone deacetylase (HDAC) inhibitor normally fermented from undigested fiber by intestinal microflora, seriously alleviated ischemia reperfusion (I/R)-induced liver injury by inhibiting the nuclear factor κB (NF-κB) pathway. The goal of this study was to investigate the effect of butyrate administrated at the onset of ischemia for HDAC inhibition in hepatic I/R injury. Sprague Dawley rats were subjected to warm ischemia for 60 min followed by 6 and 24 h of reperfusion. Butyrate was administrated at the onset of ischemia. Liver injury was evaluated by serum levels of aminotransferase, inflammatory factors, and histopathology. The levels of acetylated histone H3 and expression of heat shock protein (Hsp) 70 were measured by Western blot. After reperfusion, the levels of acetylated histone H3 significantly decreased. Butyrate treatment markedly prevented the reduction of acetylated histone H3 and upregulated the expression of Hsp70, thereby reducing liver injury. Our study demonstrated that I/R resulted in marked reduction of histone acetylation; butyrate exerted a great hepatoprotective effect through HDAC inhibition and Hsp70 induction.

## 1. Introduction

Hepatic ischemia reperfusion (I/R) injury is commonly encountered in numerous clinical conditions, including trauma, hypovolemic shock, hepatectomy, and liver transplantation. I/R can cause liver damage, organ dysfunction, and even acute and chronic rejection after transplantation, especially when using grafts from non-heart-beating donors [1,2]. Hence, minimizing I/R injury is of great clinical significance.

The pathophysiology of I/R injury includes both direct cellular damage due to ischemic insult and delayed dysfunction and damage resulting from inflammatory cascade [3]. Although our knowledge concerning the molecular and cellular pathophysiology of hepatic injury after I/R has advanced greatly, there is still no satisfying treatment available to prevent hepatic I/R injury [4].

New understandings of I/R-induced epigenetic changes suggest potential therapeutic applications. For example, global ischemia induces the deacetylation of core histone proteins H3 and H4 and dimethylation of histone H3 at lysine-9 (H3–K9) over the mu opioid receptor 1 promoter, a signature of epigenetic gene silencing [5]. In addition, kidney transplantation results in aberrant promoter hypermethylation in urine DNA, which could be used as a biomarker of acute kidney injury after transplantation [6].

It is well established that histone modifications regulate cellular processes through gene expression [7]. Histone acetylation via histone acetyl transferase (HAT) and histone deacetylase (HDAC) plays a critical role in the transcriptional activation by modulating chromatin condensation. Improper balances within histone acetylation have been shown to be associated with cancer, cardiovascular disease, and inflammatory disorders [8]. Restoring of this balance using HDAC inhibitors (HDACi) has emerged as a potential new strategy for therapeutic intervention. HDAC inhibition is already showing therapeutic promise in animal models of inflammatory bowel diseases, septic shock, airways inflammation and asthma, diabetes, cardiovascular diseases, stroke and neurodegenerative disorders [9]. The molecular mechanisms underlying HDACi protection include limiting inflammatory cell activation, reducing inflammatory factor secretion, and upregulating cytoprotectant proteins such as heat shock protein (Hsp) 70, and so on.

Butyrate is physiologically produced by the colonic bacterial anaerobic fermentation of undigested carbohydrates and fiber polysaccharides [10]. In addition to being the major energy source for intestinal epithelial cells, butyrate has recently been shown to inhibit HDAC, which is associated with gene regulation, immune modulation, cancer suppression, and so on. Potential beneficial effects of butyrate not only emerged in intestinal diseases, but also in extraintestinal ones [11].

Our previous *in vivo* study has shown that butyrate attenuated hepatic I/R injury by inhibiting nuclear factor κB (NF-κB) activation in Kupffer cells [12,13]. However, it remains unclear whether inhibition of HDAC plays an important role in the regulation of hepatic I/R injury. The present study was designed to investigate the effect of butyrate for HDAC inhibition in hepatic I/R injury.

## 2. Results and Discussion

### 2.1. Butyrate Protects against Liver I/R Injury

**Figure 1:**
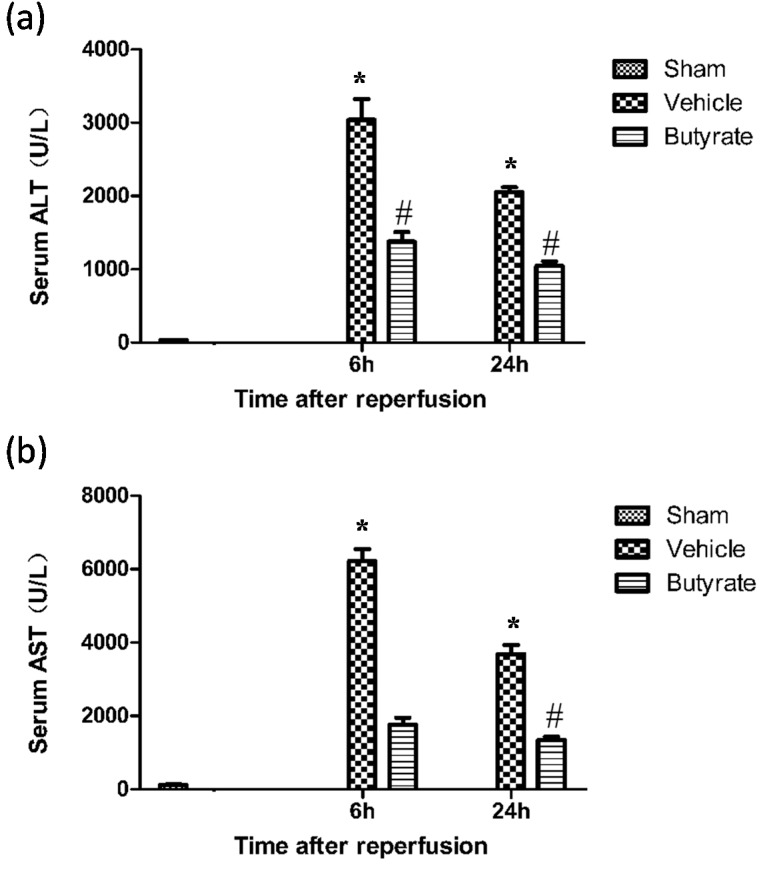
Serum alanine aminotransferase (ALT) (**a**) and aspartate aminotransferase (AST) (**b**) levels at 6 and 24 h after reperfusion. The levels of ALT and AST significantly increased in the vehicle group, which was markedly reduced by butyrate administration. Data represent means ± standard deviation (SD), *n* = 4–5 rats per group. * *p* < 0.05 *vs.* the sham group, ^#^ *p* < 0.05 *vs.* the vehicle group.

**Figure 2:**
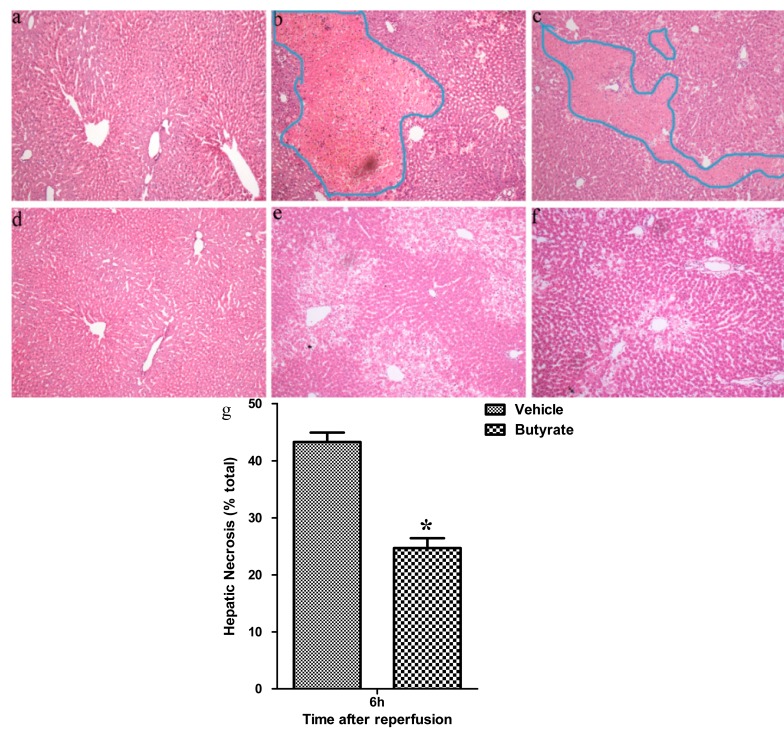
Hematoxylin–eosin (HE)-stained liver sections from the sham (**a**,**d**); vehicle (**b**,**e**); and butyrate (**c**,**f**) groups at 6 h (**a**–**c**) and 24 h (**d**–**f**) after reperfusion (original magnification: 200×), with the outlined areas in the blue line (**b**,**c**) showing hepatic necrosis area (**g**). Severe liver injury indicated by hepatocellular necrosis, sinusoidal congestion, and neutrophil infiltration was present in the vehicle group, whereas less damage was noted after butyrate treatment. * *p* < 0.05 *vs.* the vehicle group.

To assess the effects of butyrate on I/R-induced injury, rats subjected to 60 min of partial liver warm ischemia were administered with either vehicle or sodium butyrate immediately and 12 h after reperfusion. I/R resulted in a dramatic rapid increase in serum aspartate aminotransferase (AST) and alanine aminotransferase (ALT) levels in the vehicle group 6 and 24 h after reperfusion as compared with sham controls (Figure 1). Treatment with 300 mg/kg of butyrate at the onset of ischemia significantly reduced the transaminase levels following I/R, which was in agreement with our previous pretreatment reports [12] (Figure 1). Liver histopathology also confirmed the serum transaminase estimation of liver damage. Severe liver injury indicated by hepatocellular necrosis, sinusoidal congestion, and neutrophil infiltration was present in the rats following I/R. whereas less damage was noted after butyrate treatment (Figure 2).

### 2.2. Butyrate Decreases Liver I/R-Induced Inflammatory Cytokine Production and Neutrophil Infiltration

**Figure 3:**
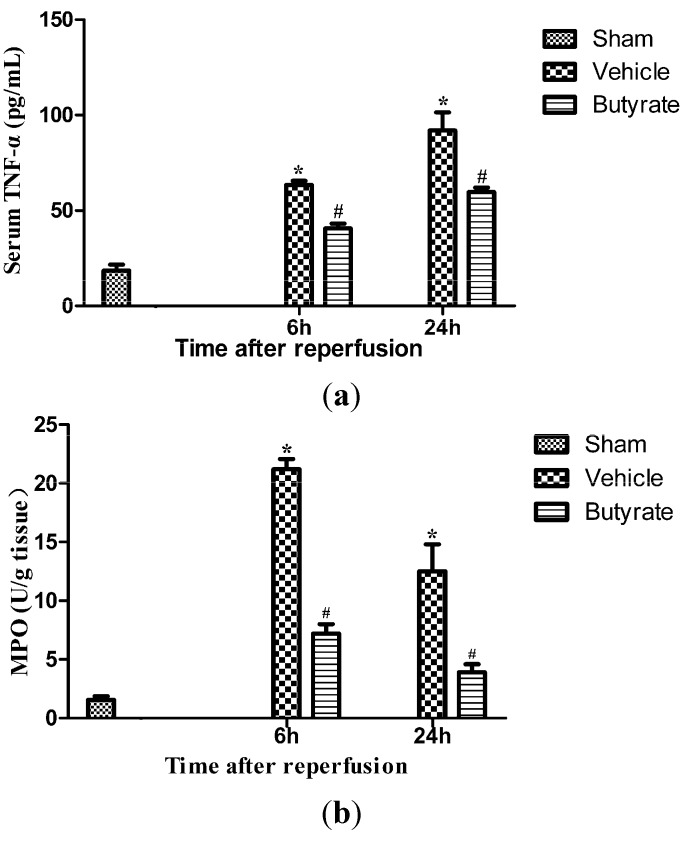
Serum tumor necrosis factor α (TNF-α) (**a**) and liver myeloperoxidase (MPO) (**b**) activities at 6 and 24 h after reperfusion. Serum TNF-α and liver MPO activities were significantly increased in the vehicle group, which were markedly reduced by butyrate administration. Data represent means ± SD, *n* = 4–5 rats per group. * *p* < 0.05 *vs.* the sham group, ^#^ *p* < 0.05 *vs.* the vehicle group.

Inflammatory cytokine production and neutrophil infiltration, which contribute to hepatic I/R injury, also reflect the degree of liver tissue damage. I/R greatly increased serum levels of tumor necrosis factor α (TNF-α), and myeloperoxidase (MPO) activity in liver tissue, which is an indicator of neutrophil infiltration (Figure 3). Butyrate significantly attenuated these inflammatory biomarkers.

### 2.3. I/R Led to Reductions in Acetylated Histones

**Figure 4:**
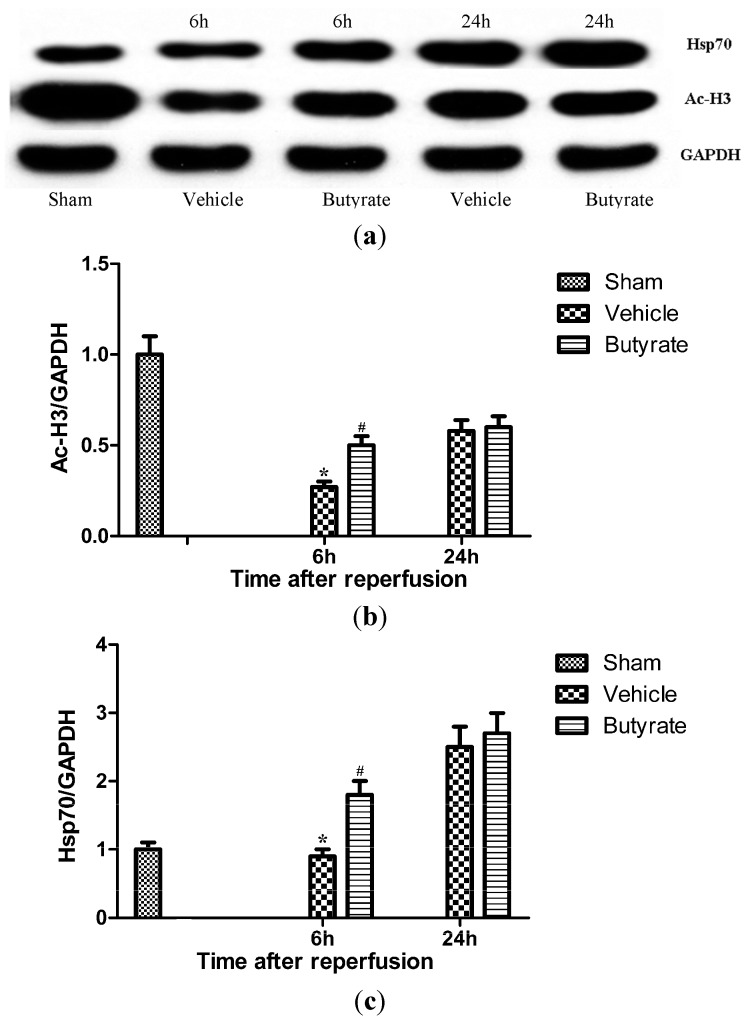
Western blot analysis of Ac-H3 and Hsp70 in liver tissue at 6 and 24 h after reperfusion. Ac-H3 and Hsp70 expression levels were measured by densitometric analysis. GAPDH expression was used as a loading control. (**a**) Blot shown is representative of three experiments with similar results; The expression of Ac-H3 (**b**) and Hsp70 (**c**) was significantly decreased in the vehicle group, which was markedly increased by butyrate administration. Data represent means ± SD, *n* = 4–5 rats per group. * *p* < 0.05 *vs.* the sham group, ^#^ *p* < 0.05 *vs.* the vehicle group.

Emerging evidence has revealed the role of acetylation in fundamental biological processes. Thus, we measured liver levels of acetylated histone H3 at Lys9 after reperfusion. As shown by Western blot analysis (Figure 4), in the vehicle group, levels of acetylated histone H3 were markedly decreased at 6 h after reperfusion, compared with the sham group, suggesting that perturbation of acetylation homeostasis is involved in I/R-induced pathological process.

### 2.4. Butyrate Promoted Histone Acetylation and Hsp70 Expression

Hepatic I/R resulted in the significant hypoacetylation of histones, whereas, butyrate treatment significantly alleviated reduction of acetylated histone levels at 6 h after reperfusion (Figure 4), compared with the corresponding vehicle group. Previous studies have reported that Hsps including Hsp70 were rapidly upregulated after reperfusion, and exerted protective action on the injured liver, brain, and heart, suggesting a correlation between Hsp70 induction and resistance to damage [7,14,15]. Moreover, histone acetylation is involved in *Hsp70* gene transcription regulation [16]. To evaluate the role of Hsp70 in mediating the hepatoprotective effects of butyrate, the levels of Hsp70 were also examined by Western blot. As expected, butyrate treatment elicited a robust increase in Hsp70 levels at 6 h after reperfusion (Figure 4), at an extent similar to acetylated histone H3 induced by itself.

### 2.5. Discussion

In this study, we showed that histone acetylation dramatically decreases in I/R liver tissues. We also demonstrated that pharmacological inhibition of HDACs with butyrate maintain histone acetylation within the ischemic liver, increased expression of hepatoprotective proteins, and afforded protection from I/R injury. Although our previous work showed butyrate pretreatment induced hepatoprotection [12], acute intervention at the onset of ischemia in this present study would be more clinically relevant.

Hepatic I/R injury is a leading cause of morbidity and mortality associated with hepatocellular dysfunction, especially after liver transplantation from non-heart-beating donors [17]. Despite major advances in the understanding of the disease etiology, effective treatment for I/R injury represents a major challenge. Given the complex pathophysiological processes involved in I/R injury, it is evident that the effective treatment strategy must target multiple pathogenetic processes induced by the I/R insult.

Chromatin remodeling by acetylation/deacetylation of histones can modulate a variety of cellular processes via regulating gene expression. Studies have shown that HATs and HDACs also target non-histone protein, representing general regulatory mechanisms in cellular processes [18]. Protein acetylation by HATs enhances gene expression via relaxation chromatin to activate transcription. However, deacetylation by HDAC induces chromatin condensation and transcriptional repression [19]. HDAC inhibitors induce histone hyperacetylation and regulate gene expression either positively or negatively, depending on the cell type [20].

Acetylation homeostasis is often disrupted in cancer, immuno-inflammatory, psychiatric, neurological, metabolic and virological disease [8]. However, the levels of acetylated histone after liver I/R are far less known. In our study, there was a significant reduction of histone acetylation in the liver at 6h after reperfusion, implying that histone acetylation may play an important role in I/R injury, which was consistent with the HDAC activity in previous study [21]. It is noteworthy that butyrate treatment immediately given after ischemia reversed hypoacetylation and exerted live protection, suggesting potential roles of histone deacetylase inhibition by butyrate in hepatoprotection. Evidence that HDAC inhibitors with molecular moieties different from butyrate also reduce I/R injury in heart and brain [7,22], corroborates the hypothesis that in addition to the inhibition of NF-κB activation, butyrate-dependent I/R hepatoprotection is causally related to HDAC inhibition. Up to now, 18 HDACs in four general classes have been identified in mammals. However, the subtype of HDAC involved in butyrate-induced hepatoprotection against cerebral infarction awaits identification.

Downstream target genes regulated by HDAC inhibitors include inflammatory genes and protective genes, such as Hsp70 [23]. Both the transcription and expression of Hsp70 are regulated and promoted by HDAC inhibitors (VPA, SB, and TSA) in *Xenopus* and *Drosophila*[16,24,25]. We found that butyrate increased the expression of Hsp70 in the live after hepatic I/R, in agreement with previous report in animal models of stroke [22]. As over-expression of Hsp70 has been shown to exert profound protective action from cerebral and hepatic I/R injury [14,26], the cellular hepatoprotective mechanism of butyrate in I/R injury is possibly associated with its upregulating of Hsp70. On the other hand, the inflammation genes were reduced by butyrate [12,13], suggestion another mechanism for hepatopretection, especially at 24 h after reperfusion, when acetylated histone H3 and Hsp70 expression have no difference in the vehicle and butyrate groups. Future study using Chromatin immunoprecipitation and microarray, will verify the candidate genes for butyrate intervention in hepatic I/R injury.

## 3. Experimental Section

### 3.1. Animals

Male Sprague Dawley rats (200–250 g) were housed in the Department of Laboratory Animal Science of Fudan University at a constant temperature of 23–25 °C and a relative humidity of 55%–60% with regular light/dark schedule. Food and water were available ad libitum. Animal handling was performed in accordance with the guidelines provided by the National Institutes of Health for the use of animals in laboratory experiments; the study protocol was approved by the Fudan University Animal Care Committee. For hepatoprotection studies, the rats were injected intravenously with 300 mg/kg sodium butyrate (Sigma, Saint Louis, MO, USA), as previously described [14], or vehicle (normal saline solution) immediately after the onset of ischemia, followed by another injection at 12 h.

### 3.2. The Warm Hepatic I/R Model

Partial warm ischemia was performed as previously described [3]. Sham-operated rats underwent the same procedure without vascular occlusion. Throughout the surgery, the body temperature was maintained at 36–37 °C with a homeothermic blanket. Rats were sacrificed at the indicated time (6 or 24 h) after reperfusion, and serum and liver samples were collected.

### 3.3. Biochemical Examination

To assess the extent of liver damage after I/R, serum levels of AST and ALT were measured in blood samples using a standard automatic analyzer (type 7150, Hitachi, Tokyo, Japan).

### 3.4. Histological Analysis

Liver tissues were fixed by immersion in 4% buffered paraformaldehyde and embedded in paraffin. Sections (4-μm) were stained with hematoxylin–eosin (HE) and assessed for inflammation and tissue damage. Quantitative analysis of hepatocellular necrosis was performed in high powerfields (200×) per each section in a blinded manner using image analysis software (Image-Pro Plus, Media Cybernetics, Sliver Spring, MD, USA). Necrotic area was outlined and expressed as a percentage of total liver area examined.

### 3.5. Western Blot

Western blot analysis was performed as previously described [15]. The primary antibodies were polyclonal rabbit antibody to Acetyl-Histone H3 (Lys9) (Cell Signaling Technology, Danvers, MA, USA), and monoclonal rabbit antibody to glyceraldehyde-3-phosphate dehydrogenase (GAPDH) (Abcam, Cambridge, UK), the secondary antibody was horseradish peroxidase-conjugated goat anti-rabbit IgG antibody (Santa Cruz Biotechnology, Dallas, TX, USA). Proteins were visualized using an enhanced chemiluminescence assay kit (GE Healthcare, Buckinghamshire, UK).

### 3.6. Statistical Analysis

Group sizes are indicated in the figure legends. Data are presented as means ± standard deviation (SD), unless otherwise noted. Statistical analysis was performed by either an ANOVA or Kaplan–Meier test. A difference of *p* < 0.05 was considered statistically significant.

## 4. Conclusions

All together, the present study furthers our understanding of the role of histone acetylation during hepatic ischemia and points to pharmacological inhibition of HDACs by butyrate as a promising strategy to reduce I/R injury.
